# Narcolepsy: a model interaction between immune system, nervous system, and sleep-wake regulation

**DOI:** 10.1007/s00281-022-00933-9

**Published:** 2022-04-21

**Authors:** Daniela Latorre, Federica Sallusto, Claudio L. A. Bassetti, Ulf Kallweit

**Affiliations:** 1 Institute of Microbiology, ETH Zurich, Zurich, Switzerland; 2 Center of Medical Immunology, Institute for Research in Biomedicine, Università della Svizzera italiana, Bellinzona, Switzerland; 3 Neurology Department, University Hospital, Bern, Switzerland; 4 Clinical Sleep and Neuroimmunology, Institute of Immunology, University Witten/Herdecke, Witten, Germany; 5 Center for Biomedical Education and Research (ZBAF), University Witten/Herdecke, Witten, Germany

## Abstract

Narcolepsy is a rare chronic neurological disorder characterized by an irresistible excessive daytime sleepiness and cataplexy. The disease is considered to be the result of the selective disruption of neuronal cells in the lateral hypothalamus expressing the neuropeptide hypocretin, which controls the sleep-wake cycle. Diagnosis and management of narcolepsy represent still a substantial medical challenge due to the large heterogeneity in the clinical manifestation of the disease as well as to the lack of understanding of the underlying pathophysiological mechanisms. However, significant advances have been made in the last years, thus opening new perspective in the field. This review describes the current knowledge of clinical presentation and pathology of narcolepsy as well as the existing diagnostic criteria and therapeutic intervention for the disease management. Recent evidence on the potential immune-mediated mechanisms that may underpin the disease establishment and progression are also highlighted.

## Introduction

Physicians have noted cases with excessive sleepiness for centuries. But the first documentations of irresistible daytime sleepiness and also of brief episodes of loss of muscle tone triggered by emotions were published only in the late nineteenth century [1–3]. In the following decades, a discussion about the entity of narcolepsy started as to whether narcolepsy was a specific disease entity or a heterogeneous syndrome (“the narcolepsies”) [4, 5]. It was in 1956 that Yoss and Daly described the classic narcoleptic tetrad of narcolepsy: excessive daytime sleepiness (EDS), cataplexy, hypnagogic hallucinations, and sleep paralysis [6]. It took until 1975 for the first consensus definition of narcolepsy, which was elaborated at the First International Symposium on Narcolepsy in France [7].

In the last decades, significant advances have been made in the understanding of the etiology, pathogenesis, symptomatology, clinical course, diagnosis, and management of the disease. Findings of hypocretin (HCRT), also named orexin, deficiency in cerebrospinal fluid (CSF), and recent data on the involvement of the immune system in narcolepsy [8, 9] opened research for new diagnostic and treatment options. Despite this progress, however, many old questions remain unanswered and still are part of current discussions [10]. The purpose of this review is to provide an update on the current knowledge of narcolepsy and to present perspectives for the future.

## Clinical presentation and epidemiology

**Fig. 1:**
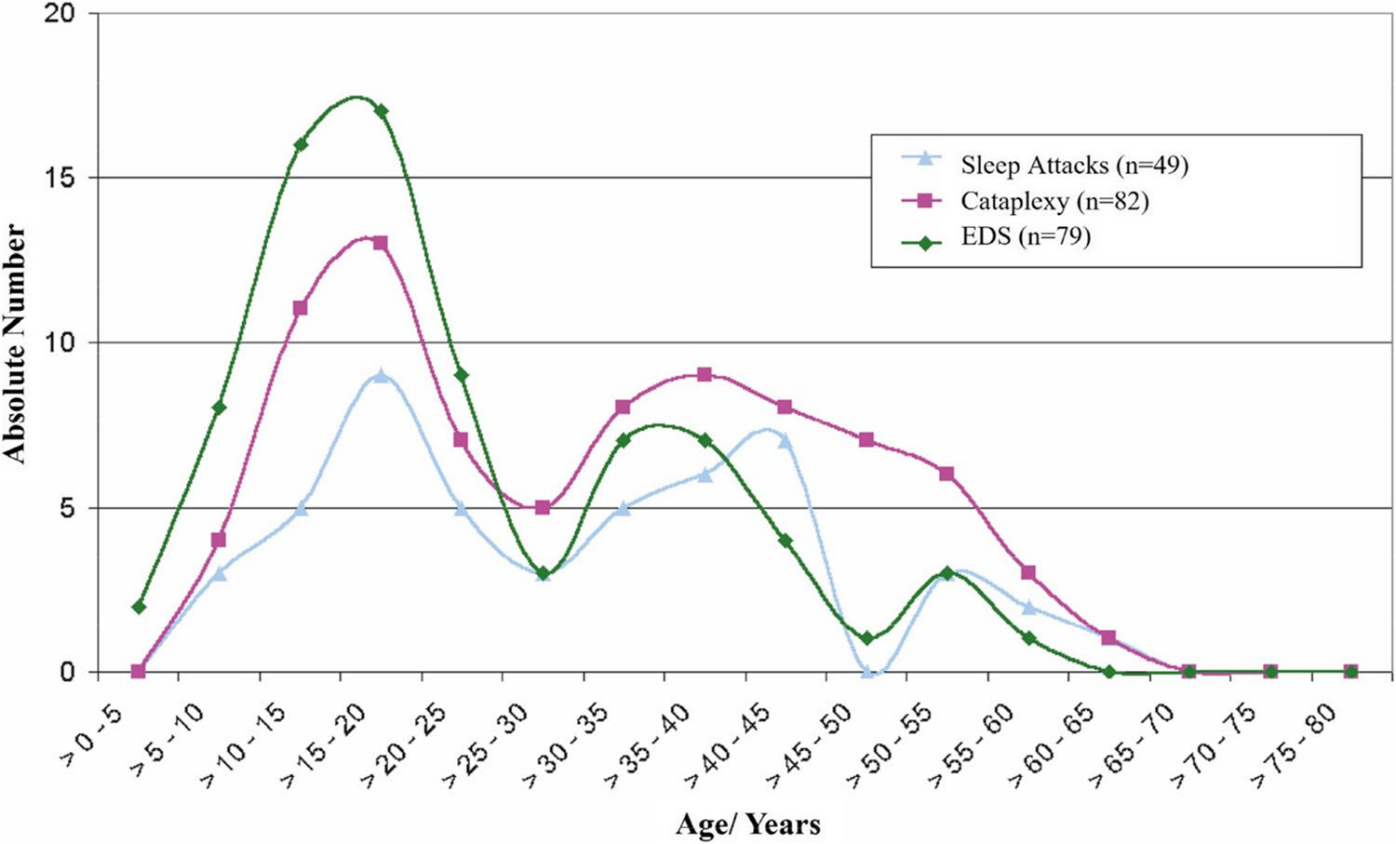
First appearance of leading symptoms of narcolepsy (sleep attacks, EDS, and cataplexy) in a German cohort. Adapted from: Mayer G, Kesper K, Ploch T, Peter H, Peter J (2002) The implications of gender and age at onset of first symptoms in narcoleptic patients in Germany – results from retrospective evaluation of hospital records. Somnologie 6:13-18

Narcolepsy is a disease of state (wakefulness-REM sleep-NREM sleep) boundary control. In almost all patients, EDS is present, whereas other symptoms can be present in different frequencies and variable combinations (see Box 1). In rare cases, symptomatic narcolepsy can manifest in patients with multiple sclerosis lesions or brain tumors [11]. Phenotypes of narcolepsy can be very diverse and include narcolepsy type 1 (NT1) with typical cataplexy that can present with decreased CSF levels of HCRT (in >90% of cases) or without any biological markers, and narcolepsy type 2 (NT2) without cataplexy but with some biological markers (such as sleep-onset REM periods). Often, narcolepsy has an acute or subacute course with symptoms developing within months or few years (< 3 years) [12]. In most cases, EDS is the first symptom and is simultaneously presenting with cataplexy (see Fig. 1). Cataplexy develops after EDS in approximately 40–60% and is only rarely present as first symptom before EDS [13]. After consolidation of symptoms, the course usually maintains stable, but also some fluctuations of all or single symptoms is possible. Fluctuations can be present on a daily, weekly, or even monthly basis. In other cases, a progressive course occurs with symptom onset separated by several years, sometimes more than 30 [14].

**Box 1** Clinical presentation of narcolepsy

**Table:** 

Key symptoms	
- Excessive daytime sleepiness incl. sleep attacks, involuntary napping, automatic behavior)	
- Concentration and memory deficits	
- Fatigue incl. lack of energy	
- Cataplexy (partial and complete)	
Further symptoms	
- Disrupted nocturnal sleep	
- Nightmares, vivid dreaming, enactment of dreams	
- Sleep paralysis	
- Sleep-related hallucinations (visual, auditory, tactile)	
- Overweight, weight gain at disease onset	
- Autonomic disturbances	
Frequently associated symptoms	
- Sleep disorders (Restless legs syndrome, sleep apnea, parasomnias)	
- Depression, Anxiety	
- Attention-deficit hyperactivity disorder	
- Headaches, Migraine	

Narcolepsy has a prevalence of 20–50 per 100,000 individuals, depending on the method of assessment, the applied diagnostic criteria, and also on countries and populations, and can be therefore considered a rare disease [15–17]. Data on the incidence of narcolepsy vary between 0.7 and 1.4 per 100,000 individuals per year [18, 19]. Some studies indicate a slight dominance of males being affected by narcolepsy. In contrast, in children and adolescent females may be slightly more often affected [19]. Basically, narcolepsy can affect individuals at any age. A first symptom of narcolepsy, which is EDS usually, in most cases presents during adolescents or young adulthood [19]. In 10–15% of cases, symptoms even begin before the age of 10 years [20].

### Daytime symptoms

Excessive daytime sleepiness (EDS) is present in all narcolepsy patients as an involuntary napping/sleeping “against one’s own will” [12, 21]. Falling asleep is more likely in monotonous, passive situations, e.g., as passenger in a car, but can also take place while being active. Nap duration can be short (seconds to few minutes) or sometimes also long (hours). Often naps with durations of 15–20 min are felt to be best helpful [21, 22]. There is also a chronic (daily) feeling of sleepiness. This feeling is severe and often only physical or mental (exciting) activities can suppress EDS for a short time. Another symptom is “automatic behavior.” This refers to an incorrect and inattentive implementation of a behavior, such as putting shoes in the refrigerator. Usually, patients do not remember this episode and “wake up” at one certain point of time. Automatic behavior hence represents some kind of microsleep, and again an in-between state (wake-sleep). Another daytime symptom is fatigue (in up to 60%), which refers to the complaint of physical and/or mental exhaustion with difficulties in daily activities. Increased rest or sleep does not significantly improve fatigue. Fatigue can also have a negative impact on EDS [23]. EDS can also cause or being associated with other symptoms such as visual problems, e.g., blurred vision, headache, and migraine or concentration and memory deficits (see also below).

### Cataplexy

Cataplexy is pathognomonic for narcolepsy. It is defined by a sudden, bilateral and only temporary loss of muscle tone, usually triggered by emotions and with simultaneously preserved consciousness. Cataplexy often has a certain sequence, starting cranial with loss of muscle tone in the face and neck, and then moving downwards affecting the body, arms and legs. The normal state of consciousness is important for the differentiation from other symptoms, such as epileptic seizures or syncope. Most frequently, positive emotions such as laughter can trigger cataplexy. Other emotions such as surprise, anger or fear may also be triggers [24]. Cataplexy is more often occurring when EDS is present strongly. In children, cataplexy can present with some additional features, such as hyperkinesias or tongue protrusion [25]. In most NT1 cases, cataplexy becomes present within 4 years after onset of EDS. In a section of patients initially diagnosed as NT2, cataplexy evolves after many years (up to 40 years).

### Sleep paralysis and sleep-related hallucinations

Sleep paralysis (SP) refers to the inability to move any voluntary muscle (hence also speaking is not possible) while being awake and having preserved consciousness. SP usually occurs entering sleep or when waking up, particularly. SP is often frightening, especially the first times happening. SP usually immediately stops when being touched by someone. Hallucinations and sleep paralysis can present together or independently. Hallucinations occur at sleep onset (hypnagogic), on awakening (hypnopompic), or also during daytime naps. Sometimes a differentiation between hallucination, dreaming, and wakefulness is difficult. Hallucinations often are visual but also include other senses such as olfactory, auditory, haptic, or “spiritual” [26].

### Nocturnal sleep

Nocturnal sleep is often poor in narcolepsy and is fragmented with several wake periods which are often short but can also last hours [27]. In narcolepsy, intensive/vivid dreaming and also nightmares are frequent. In 25–75% of persons affected by narcolepsy, enactment of dreaming, classified as “REM sleep behavior disorder” (RBD) parasomnia, is present [28]. Other causes affecting nocturnal sleep are co-existing obstructive sleep apnea, restless legs syndrome, and periodic limb movements during sleep [29–32]. After waking up in the morning, wakefulness is established quite fast and lasts for some hours until EDS occurs. It should be noted however that nocturnal sleep duration, sleep periods during daytime, and 24-h sleep duration do not vary much in narcolepsy patients compared to healthy individuals. There is only a very weak correlation between sleep fragmentation and EDS in narcolepsy [33, 34]. In children and rarely in adults, long sleep duration and undisturbed sleep is described. In adults, this may lead to re-consider the diagnosis, as long (> 10 h) and undisturbed sleep, and sleep inertia are typical features of idiopathic hypersomnia [35].

### Cognitive disturbances

Almost all patients describe some or major difficulties with concentration, attention, and memory [36–38]. In particular higher-order cognition, decision-making, and emotional processing are affected [39]. Cognitive deficits may be the key-limiting symptom in some patients and should be addressed. Deficits are thought to be associated or due to EDS. Some other data indicate for a potential association between the HCRT loss and neurodegeneration, causing cognitive deficits [40]. Attention deficits and diagnosis of attention deficit (hyperactivity) syndrome are frequent in narcolepsy [41]. Misdiagnosis may occur, as awareness about cognitive deficits in narcolepsy is low.

### Other features

Several other features can occur in narcolepsy. Psychiatric disorders, in particular depression and anxiety are frequent (20–30%) [42]. They are most probably cause of narcolepsy symptoms and their resulting limitations in performance, social interaction and quality of life [43, 44]. Metabolic alterations are also found, weight gain and obesity in particular. Abnormal eating behavior and nocturnal eating are also common [45, 46], while autonomic disturbances such as skin temperature dysregulation and gastric disturbances are less frequent [47].

## Pathology

HCRT has been discovered in 1998. *HCRT* gene is exclusively expressed by approx. 70,000 neurons located in the lateral hypothalamus, consecutively named “HCRT-neurons”. *HCRT* gene encodes a single polypeptide, prepro-HCRT that then forms the two peptides HCRT-1 and HCRT-2. HCRT-1 and HCRT-2 activate two specific receptors (*HCRT-1* and *-2* receptor). These are widely distributed across the whole brain and involved in various functions including sleep and wakefulness, stability of the sleep/wake states, arousal, feeding, reward, fear, anxiety, and several cognitive functions [48].

In the year 2000, a selective reduction of HCRT-neurons (and axons) in the lateral hypothalamus has been found in human narcolepsy [49, 50]. In autopsy series of narcolepsy affected individuals, the loss of neurons was approx. 90% (75–95%). It was a selective loss of HCRT-neurons since other neurons in the lateral hypothalamus, such as the MCH (melanin-concentrating hormone)-producing neurons, directly neighboring the HCRT-neurons, were not affected. The brain tissues showed an elevated level of gliosis but no inflammatory or neurodegenerative changes. The loss of HCRT-neurons and elevation of glial fibrillary acidic protein (GFAP) staining were maximal in the posterior and tuberomammillary hypothalamic region [51]. Data from animal studies suggest that a reduction of > 80% of HCRT in the CSF, consistent with the loss of virtually all HCRT-neurons, would result in the symptoms of narcolepsy, such as the lead symptoms EDS and cataplexy [52]. A partial loss of HCRT-neurons leads instead to EDS (narcolepsy) without cataplexy and with normal CSF HCRT levels. Along this line, analysis of postmortem brain from patients with narcolepsy without cataplexy (*n* = 2) showed that one had a normal number of HCRT-neurons, and one had a 33% decrease in HCRT-neurons [53]. All these observations led to the assumption of narcolepsy being merely due to the selective HCRT-neuronal cell disruption and loss. Notably, a new report has recently challenged this model showing that HCRT-neurons are still present in the brain of animal models and narcolepsy patients, but HCRT gene is epigenetically silenced in these neurons [54].

In the last decade, an increased number of histamine neurons have been identified in the tuberomammillary nucleus (TMN) in NT1, and also in mouse models of narcolepsy. Increase of neurons was > 50% compared to healthy individuals [55]. If this overexpression is part of the pathogenesis of narcolepsy or a compensatory mechanism due to the HCRT-neuronal loss remains unclear. Although the HCRT system is crucial for narcolepsy, circuit perspectives of the pathophysiological mechanisms encompassing brain regions, neuronal circuits, cell types, and transmitters have been suggested, recently [56].

## Etiology and immunity

Narcolepsy is suggested to be the results of an autoimmune process occurring in genetically predisposed individuals upon triggering by environmental factors that would lead to a selective loss of HCRT-neurons in the lateral hypothalamus [57].

### Genetic predisposition

A familial predisposition in narcolepsy has long been recognized [58]. Genome-wide association studies have revealed the strongest HLA gene polymorphism association ever described with up to 98.5% of NT1 patients and 40–60% of NT2 patients carrying the HLA-DQB1*06:02 allele [59–61]. The HLA-DQB1*0602 allele is present in 20–30% in the general population of Western countries.

Polymorphisms in additional immune-related genes, such as those that encode MHC class I molecules, T cell receptor alpha (TCRα) chain (*TRA*), P2Y purinoceptor 11 (*P2RY11*), tumor necrosis factor ligand super family member 4 (*TNFSF4*), and cathepsin H (*CTSH*) have also been shown [62–67]. Many of these molecules are involved in antigen processing and presentation as well as in antigen recognition by T lymphocytes. They may act by influencing the selective presentation of particular self-antigens to T cells and by shaping the T cell repertoire, thus suggesting that T cells may play a key role in the disease.

### Environmental factors

Disease concordance rate in monozygotic twins is 25–31%, which indicates that environmental factors have an important role besides genetic predisposition in narcolepsy [68]. Bacterial and viral infections have been associated with recent-onset narcolepsy cases. The identification of elevated titers of anti-streptococcal antibodies (Abs) in NT1 patients within 1 year of onset suggested an association with preceding *Streptococcus pyogenes* infections [69]. However, the most striking association has been described in 2009–2010 after the H1N1 influenza pandemic that affected mostly Asian countries. A retrospective analysis of narcolepsy onset cases in China (1998–2010) showed seasonal patterns correlating with H1N1 influenza infections [70]. Notably, they found a 3-fold increase in narcolepsy incidence after the 2009 H1N1 influenza pandemic, which returned to pre-pandemic basal levels in later years [70, 71]. Along this line, European countries experienced 6-fold to 9-fold increase in the incidence of narcolepsy, especially in children, following the 2009–2010 Pandemrix vaccination campaign that was carried out to prevent pandemic H1N1 influenza [72–76]. However, this evidence was not confirmed with any other influenza vaccines, which may be explained by the vaccine composition itself [77]. Importantly, a recent analysis of 508 newly diagnosed NT1 cases identified a new peak in children/adolescents (i.e., 2.09-fold increase) in 2013, which likely was not related to pandemic H1N1 influenza infections or vaccination, but rather associated to another undefined environmental factors [78]. Small case series and case reports also indicate that other vaccines may have an impact on the evolution of narcolepsy [79].

### Autoimmune responses in narcolepsy

Autoimmune diseases are chronic disorders that develop due to the breakdown of immune tolerance towards self-antigens, which would result in the development of auto-Abs and/or autoreactive T cells. Given the strong association with polymorphisms in genes involved in immune regulation as well as the identification of potential environmental triggers, for long scientists have attempted to demonstrate the autoimmune basis of narcolepsy.

### Autoantibodies and autoreactive T lymphocytes

The search for the presence of auto-Abs in the serum of narcolepsy patients has led so far to inconclusive results. In 2010, three independent research groups identified increased titers of auto-Abs directed against the intracellular protein tribbles homologue 2 (TRIB2) in narcolepsy patients with recent onset compared to controls [80–82]. Later, additional self-antigens, including the neuropeptide glutamic acid-isoleucine/a-melanocyte-stimulating hormone (NEI/aMSH) [83], the hypocretin receptor 2 (HCRTR2) [77], prostaglandin receptor D2 [84], and neurexin-1-alpha (NRXN1) [85], have been identified as targets of auto-Abs in narcolepsy patients. However, many of these self-antigens are found not only in HCRT-neurons but also in other cell types both in the CNS and in the periphery [86–89]. Moreover, some of the described auto-Abs were also detected in sera from patients with other sleep disorders and in healthy controls and not found in other studies on narcolepsy patients [90–95]. Overall, this evidence suggests that auto-Abs are not primarily involved in HCRT-neuronal disruption but may rather play a secondary role in a subset of patients.

A low-grade inflammatory milieu in the blood and CSF as well as systemic changes in the distribution and activation of blood-circulating T cell populations have been described in NT1 patients [96–103]. Despite these observations pointed to T cells as key players in the disease immunopathology, investigations provided for long only weak indications of the existence of autoreactive T cells in these patients. This suggests that, if existing, self-reactive T cells would mostly likely be confined in the brain where the cognate antigen is present and circulate in the blood only at very low frequency, thus making challenging their detection with traditional methods [104–107]. However, the employment of more sensitive experimental approaches in recent years resulted critical to shed light on this aspect. Notably, several reports have shown the existence of autoreactive T cells directed against antigens expressed by HCRT-neurons in NT1 patients [9, 107–110]. We optimized an unbiased approach, based on the combination of T cell libraries, ex vivo T cell stimulation and TCR sequencing, which led to the identification of rare autoreactive T cells in the blood and CFS of narcolepsy patients. The study detected memory CD4^+^ and CD8^+^ T cells reactive to the neuronal antigens HCRT and TRIB2 in all 19 patients analyzed. Although their frequency resulted very low (range < 1–89.7 cells per 10^6^ cells), it was shown to be significantly increased in NT1 patients compared to HLA-matched healthy donors [9]. Surprisingly, HCRT-reactive memory CD4^+^ T cells were also found in the few HLA-DQB1*06:02 negative NT1 patients analyzed as well as in NT2 patients, thus suggesting that a broader spectrum of sleep-related disorders may underlie an autoimmune basis [9, 111]. Moreover, we performed an in-depth characterization of a large number of autoreactive CD4^+^ T cell clones revealing that the T cell response is directed against several epitopes of HCRT and TRIB2 antigens and is restricted primarily to HLA-DR molecules [9].

Follow-up studies employed the tetramer approach to search specifically for HCRT-reactive CD4^+^ T cells restricted to HLA-DQ molecules in the blood of NT1 patients, confirming the existence of these cells at increased frequencies compared to HLA-matched healthy donors [108, 109]. Luo et al. also showed that the autoreactive T cell response is mostly directed against the C-amidated version of the HCRT peptides, a post-translational modification necessary for their biological activity corresponding to the naturally processed/secreted HCRT-1 and HCRT-2 [108, 112]. Finally, several reports analyzing the TCR repertoire of autoreactive CD4^+^ T cells indicated that these cells are expanded in the blood of NT patients and, importantly, revealed the existence of autoreactive clones bearing public CDR3 motifs in their TCR in different NT1 patients [9, 108, 109, 113]. The usage of TRAJ24 rearrangement was found enriched in HLA-DQ-restricted HCRT-specific CD4^+^ T cells, which is in line with the known association of *TCRA* polymorphisms to the disease [108, 109]. These results indicate that rare CD4^+^ T cells directed against HCRT-neurons are shared and expanded in the blood of NT1 patients, thus pointing to their direct contribution to the disease immunopathology. However, the relative involvement of self-reactive CD4^+^ T cells recognizing HCRT-neuronal antigens in association with HLA-DR and HLA-DQ molecules remains to be defined.

The generation of transgenic mice expressing the influenza viral antigen hemagglutinin (HA) in HCRT-neurons provided crucial information on the immune-mediated mechanisms underlying the disease establishment. These mice showed selective neuronal loss and developed narcolepsy-like symptoms only when both HA-specific CD4^+^ and CD8^+^ T cells were adoptively transferred. While CD4^+^ T cells were able to infiltrate the brain and promote local inflammation but did not induce the disease, CD8^+^ T cells mediated neuronal damage and promoted the establishment of narcolepsy-like symptoms in this mouse model [114]. Along this line, Pedersen at al. searched specifically for autoreactive CD8^+^ T cells in the blood of NT1 patients. To this end, the authors used DNA barcode-labelled MHC class I multimers loaded with a library of 1183 self-peptides derived from seven proteins with preferential expression in HCRT-neurons [110]. Their work demonstrated increased frequencies as well as broader reactivity of blood circulating self-reactive CD8^+^ T cells compared to healthy donors. These data confirmed findings from our previous study, in which HCRT-specific CD8^+^ T cells were also detected but resulted less frequent than HCRT-specific CD4^+^ T cells in the blood of NT patients [9]. Importantly, HCRT-specific CD8^+^ T cell clones were also isolated from the CSF, thus strongly supporting evidence from in vivo studies of a CD8^+^ T cells infiltration in the brain and direct killing of HCRT-neurons in human narcolepsy [9].

Most of the reports described above have focused on NT1 patients who suffered from the disease for many years, thus some important information on the ongoing response at early stage of the disorder may have been overlooked. Some insights on this issue came from the analysis of NT2 patients and children with recent narcolepsy onset [9, 107]. CD4^+^ and CD8^+^ T cells directed against HCRT were found at similar frequencies and showed higher magnitude of response in the blood of NT2 patients compared to NT1 patients. Notably, potentially pathogenic HCRT-specific CD8^+^ T cells were isolated from the CSF of one NT2 patient that developed full blown NT1 during the study [9]. Moreover, in another study [107], HCRT-reactive T cells were easily detectable with traditional methods in the blood of children with recent narcolepsy onset, thus indicating that in early phases of the disease self-reactive T cells may be expanded in vivo.

Overall, these observations point to a key role of autoreactive CD4^+^ and CD8^+^ cells in narcolepsy. However, new investigations are needed to better define the relative involvement of autoreactive immune responses at different stages of the disease establishment and progression. Notably, the SPHYNCS study, a multicenter cohort study that is currently ongoing in Switzerland with the main goal to provide a systematic evaluation over time of clinical and immunological features in NT1, NT2 and patients suffering from other hypersomnolence disorders, may hopefully shed light on this aspect in the future [111].

### Molecular mimicry as potential trigger of narcolepsy

Previous exposure to infections or vaccination is considered a potential trigger of the aberrant immune response in narcolepsy that could act either through a mechanism of molecular mimicry or by enhancing the immune response against self-antigens. So far, attempts to identify a vaccination-associated pathogenic signature in blood-circulating immune cells or increased titers of auto-Abs targeting exclusively HCRT-neurons in post-Pandemrix NT1 have failed [91, 99]. However, these patients were found to have an increased production of the pro-inflammatory cytokine IFN‐γ upon in vitro stimulation of peripheral blood mononuclear cell (PMBCs) with the self-antigen protein-O-mannosyltransferase 1 (POMT1) [105] as well as augmented frequencies of auto-Abs directed against the self-antigens monosialodihexosylganglioside (GM3) and POMT1 [105, 115]. Given that GM3 and POMT1 are expressed also in the peripheral nervous system, how the altered immune response observed in post-Pandemirx NT1 patients may represent the causal effect of the disease establishment remains obscure.

Nevertheless, the identification of sequence similarity of HCRTR2 and HCRT itself with nucleoprotein (NP) and HA of 2009 H1N1, respectively, strongly suggested a molecular mimicry mechanism in the pathophysiology of narcolepsy [77, 108]. The existence of a cross-reactive immune response in NT1 patients is however still unclear. Interestingly, in 2015 Ahmed et al. described increased titers of auto-Abs against HCRTR2 that in competition ELISA assay resulted potentially cross-reactive to NP of 2009 H1N1 in the sera of NT1 patients with a history of Pandemrix vaccination [77]. However, following reports have not confirmed these findings [90, 93]. Moreover, HCRT- and TRIB2-specific CD4^+^ T cell clones isolated from a cohort of non-vaccinated NT patients did not show cross-reactivity to influenza antigens in in vitro screenings [9]. Contrarily, bulk TCR sequencing analysis of CD4^+^ T cells reactive to HCRT_NH2_-tetramers or to pHA_273–287_-tetramers identified a few clones bearing shared TCR CDR3 sequences [108], thus further supporting a cross-reactive T cell immunity between self-antigens and viral antigens in narcolepsy. However, this evidence has yet to be confirmed in independent studies as well as in in vitro functional assays of single T cell clones. Of note, potential cross-reactive CD4^+^ T cell clones were found in HCRT- or HA-reactive T cell fractions isolated both from controls and NT patients independently on whether they were previously vaccinated with Pandemrix [108], thus challenging the model of a pathogenic role of these cells in narcolepsy.

### Current model of narcolepsy immunopathology

**Fig. 2:**
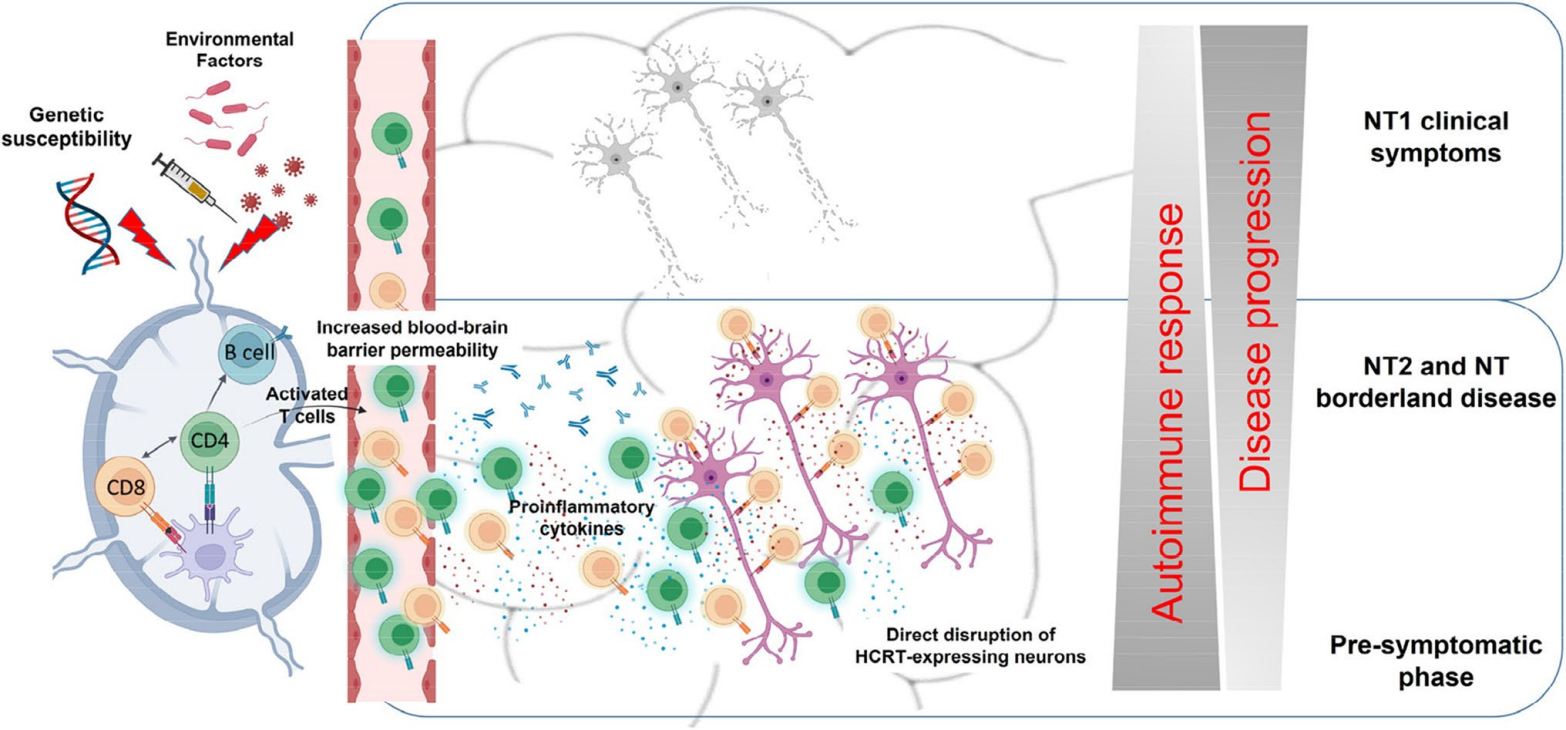
Immune-mediated mechanisms and disease progression in narcolepsy (Created with BioRender.com)

Converging lines of evidence from studies in humans and animal models strongly reinforce the notion of a T cell contribution to the pathophysiology of narcolepsy [107–110]. Narcolepsy may be a progressive disorder developing in genetic predisposed individuals upon single or combined exposure to certain environmental factors that would trigger an immune attack to HCRT-neurons by a molecular mimicry mechanism. Autoreactive CD4^+^ T cells may take part to the immunopathology in the initial phases of the disease by promoting a local inflammatory environment in the CNS, which would in turn induce the expression of MHC class I molecules on neurons as well as alter the blood–brain barrier integrity with consequent recruitment of other immune cell types. Cytotoxic self-reactive CD8^+^ T cells may then also reach the CNS where they would recognize the cognate antigens on MHC class I molecules on HCRT-neurons, thus resulting in their selective disruption (see Fig. 2). The inflammatory environment may also promote epigenetic changes in HCRT-neurons that would progressively lead to HCRT gene silencing and reduced production of HCRT [54]. In this model, sustained or repetitive autoimmune responses may initiate many years before any signs of narcolepsy symptomatology and would contribute to the disease progression, thus leading to the development of full-blown NT1 symptoms only at later stage, when more than 80–90% of HCRT neurons are lost or silenced (Fig. 2) [49, 50, 54]. While the disease would develop over time due to the progressive immune-mediated neuronal disruption and/or HCRT gene silencing with consequent impairment in HCRT expression, the autoimmune response would decrease due to the decline in antigen availability.

## Diagnosis

Currently, mean latency between first symptom onset, which is usually EDS, and diagnosis is often >5 years in Europe [21]. Narcolepsy hence is often overseen or misdiagnosed. There is a great need to improve diagnosis, both in speed, and in accuracy. Current diagnostic criteria (ICSD-3) include clinical history and symptomatology, sleep examinations, and laboratory features [116]. Based on the presence of cataplexy and HCRT levels in the CSF, ICSD-3 can differentiate between NT1 and NT2 (see Table 1).

**Table 1:** ICSD-3 criteria for the diagnosis of narcolepsy

Type	Type 1 narcolepsy	Type 2 narcolepsy
Clinical symptoms	Chronic, excessive daytime sleepiness (EDS) > 3 months	
	Cataplexy (> 1x)	
Sleep examinations	> 1 SOREM periods on MSLT (or PSG); mean sleep latency < 8 min on MSLT	
CSF (in addition to or instead of sleep examinations	Hypocretin deficiency (< 110 pg/ml)	
	Exclusion of other causes for EDS (i.e., brain MRI)	

*SOREM* sleep onset REM, *MSLT* Multiple Sleep Latency Test, *PSG* polysomnography, *CSF* cerebrospinal fluid

The option only to make a diagnosis based upon clinical features and HCRT levels in CSF are formally possible, but in order to exclude other causes for EDS (e.g., sleep apnea) and due to methodological and availability difficulties in the measurements of HCRT levels in CSF, sleep studies are mandatory for diagnosis.

The differential diagnosis of the narcolepsies includes mainly other disorders with the lead symptom of EDS or excessive need for sleep (ENS), defined as “hypersomnolence disorders.” The narcoleptic “borderland” includes idiopathic hypersomnia (IH), hypersomnia associated with psychiatric, neurological and medical disorders, chronic sleep deprivation, but also “idiopathic EDS”. The differentiation of NT2 and IH or idiopathic EDS is often very difficult, in particular. Red flags for narcolepsy can be seen in Box 2.

There has been some criticism on the diagnostic criteria and a revision has been suggested. The creation of a more consistent, complaint driven, hierarchical classification, containing levels of certainty have been suggested [10].

**Box 2** Red flags narcolepsy

**Table:** 

i. Differentiation of EDS (e.g., sleeping “against the own will”, chronic) from tiredness, fatigue, depressive mood or attention deficits.	
ii. EDS has an impact on daytime activity, workability and quality of life	
iii. Cataplexy very specific for narcolepsy (“weak with laughter”)	
iv Poor nocturnal sleep with vivid dreaming, nightmares, and/or enactment of dreams, in young adults in particular	

## Management

Treatment of narcolepsy includes counseling, self-care, behavioral strategies, psychological treatment, and pharmacological therapies [117, 118]. Patient often state on the importance and preference of non-pharmacological therapies [118].

### Non-pharmacological approaches

Firstly, the patient should be informed about the etiology, pathogenesis, course of the disease and available and future management options. Also, working, driving and social aspects have to be addressed. Second, self-care and behavioral strategies and recommendations should be described. Strongest recommendations are made for scheduled napping during daytime [118]. Usually short naps, e.g., 10–15 min, are helpful and felt to be refreshing. In some patients, in children and often in the afternoon, also longer naps of 30–45 min may be even more appropriate [118, 119]. A regular sleep-wake rhythm is also recommended. Some data also indicate for an improvement of EDS by low-carb diet and by regular physical exercise. Caffeine may be used in a reasonable quantity [120, 121]. If possible, patients should first implement strategies mentioned for at least 4 weeks in order to experience the impact of these therapies.

Most patients benefit from exchange with other patients affected by narcolepsy. Potential points of contact with self-help groups should be provided [118]. Psychological counseling or cognitive behavioral therapy is also needed is patients with a co-morbid or reactive affective disorder, depression in particular. Subjects of psychotherapeutic interventions often include coping, daily structure, emotion regulation and depression.

### Pharmacological therapy

Currently available pharmacological therapy is purely symptomatic and focuses on lead symptoms of narcolepsy: EDS and cataplexy (Table 2) [117, 118].

**Table 2:** Frequently used medicines for narcolepsy (in adults): compound, mechanism of action, maximum dosage per day, half-life, and EMA approval text

Drug (name)	Mechanism of action	Max. dosage per day	Half-life (h)	EMA approval (official text) Therapeutic indications
Modafinil	Dopaminergic	400 mg	11–15 h	Treatment of excessive sleepiness associated with narcolepsy with or without cataplexy in adults
Pitolisant (Wakix)	Histaminergic (increase of CNS histamine)	36 mg	10–12 h	Treatment of narcolepsy with or without cataplexy in adults
Sodium oxybate (i.e., Xyrem)	GABAergic	9 g	3–4 h	Treatment of narcolepsy with cataplexy in adult patients.
Solriamfetol (Sunosi)	Dopaminergic/norepinephrinergic	150 mg	7.1 h	To improve wakefulness and reduce excessive daytime sleepiness in adult patients with narcolepsy (with or without cataplexy)
Venlafaxine^3^ (SSRI-antidepressant)	Serotoninergic (and noradrenergic)	For cataplexy usually not higher than 150 mg/day	15 h	Off-label
Clomipramine^2^ (tricyclic antidepressant)	Norepinephrinergic/serotoninergic	For cataplexy usually not higher than 50mg/day	21 h	Off-label
Methylphenidate	Dopaminergic	60 mg	3 h Depend if IR or XR	Off-label (only in few European countries for EDS in narcolepsy)
Dexamphetamine^1^ (as example for amphetamine-derivates)	Norepinephrinergic (dopaminergic)	60 mg (morning and/or morning—noon, or on demand)	11 h	Off-label

^1^For other amphetamines to be checked individually

^2^For other tricyclics to be checked individually

^3^For other SSRIs/SNRIs to be checked individually

## Future directions

Although substantial increase in the understanding and management of narcolepsy has been made in the past decade, several issues remain open. Narcolepsy is a complex immune-mediated brain disorder, and additional information about the mechanisms of the etiopathology is needed.

Narcolepsy is a rare disease and a long delay in the diagnosis is associated with the development of psychiatric disorders and socio-economic disadvantages. A better awareness of narcolepsy, all in the general population, general practitioners and also sleep specialists is needed. New drugs have been approved and have extended available treatment options. Nevertheless, further medication is needed, as most patients are not (fully) satisfied with the management. Currently treatments that include the HCRT system, such as HCRT receptor agonists are under investigation [122]. If immune-based therapies, applied in a very early stage of the disease, may result in a modulation or stopping of the autoimmune process, remains unclear. Published data, mainly case reports, do no support this hypothesis until now.
