# Correction to: Narcolepsy: a model interaction between immune system, nervous system, and sleep-wake regulation

**DOI:** 10.1007/s00281-022-00946-4

**Published:** 2022-05-26

**Authors:** Daniela Latorre, Federica Sallusto, Claudio L. A. Bassetti, Ulf Kallweit

**Affiliations:** 1 Institute of Microbiology, ETH Zurich, Zurich, Switzerland; 2 Center of Medical Immunology, Institute for Research in Biomedicine, Università della Svizzera italiana, Bellinzona, Switzerland; 3 Neurology Department, University Hospital, Bern, Switzerland; 4 Clinical Sleep and Neuroimmunology, Institute of Immunology, University Witten/Herdecke, Witten, Germany; 5 Center for Biomedical Education and Research (ZBAF), University Witten/Herdecke, Witten, Germany

**Correction to: Seminars in Immunopathology**

10.1007/s00281-022-00933-9

In the original version of this article, the given and family names of Federica Sallusto were incorrectly structured.

The original article has been corrected.

